# Serotoninergic antidepressants combination in psilocybin-assisted psychotherapy: a case report

**DOI:** 10.3389/fpsyt.2024.1394962

**Published:** 2024-07-16

**Authors:** André Do, Vanessa Michaud, Jean-François Stephan, Miltiadis Moreau, Élise Benoît, Félix-Antoine Bérubé, Antoine Bibaud-De Serres, Alain Taillefer, Philippe Vincent

**Affiliations:** ^1^ Department of Psychiatry, Université de Montréal, Montreal, QC, Canada; ^2^ Faculty of Pharmacy, Université de Montréal, Montreal, QC, Canada

**Keywords:** psilocybin-assisted psychotherapy, difficult-to-treat depression, serotoninergic antidepressants, antidepressants combination, case report

## Abstract

Psilocybin has reemerged as a promising treatment for difficult-to-treat depression (DTD). Although there is limited evidence regarding interactions between psilocybin and other psychotropic drugs, clinical trials require that patients discontinue their antidepressants before study entry to isolate the benefits of psilocybin and to minimize the risk of adverse events. We present the first case of an adult patient with DTD who received psilocybin-assisted psychotherapy (PAP) in combination with two serotoninergic antidepressants (duloxetine and vortioxetine). Since he displayed a partial response after the first PAP session, he agreed to discontinue duloxetine (but refused to stop vortioxetine) before the second PAP session to see if it could improve the therapeutic efficacy of psilocybin. However, his anxiety and depressive symptoms worsened. Psilocybin was well-tolerated in both PAP sessions; mild headaches were the main adverse effects experienced by the patient, and there were no cardiovascular safety concerns. This case report suggests that serotoninergic antidepressants combination with psilocybin appears to be safe and that antidepressant discontinuation prior to PAP may not be necessary. Since the continuation of antidepressants during PAP has the potential to improve treatment acceptability and accessibility, future research should assess whether psilocybin can be administered concurrently with antidepressants.

## Background

In recent years, psilocybin has reemerged as a promising treatment for difficult-to-treat depression (DTD) (1). Currently, clinical trials require that patients discontinue their antidepressants before study entry both to isolate the therapeutic effects of psilocybin and to minimize the risk of severe adverse events, such as serotonin syndrome. However, there is limited evidence regarding drug-drug interactions with psilocybin and other psychotropic drugs commonly used in depression (2). Given the challenges associated with discontinuing antidepressants, this raises the question whether stopping them prior to psilocybin-assisted psychotherapy (PAP) is necessary. Here, we report the first case of an adult with DTD who received 25 mg psilocybin with psychological support, in combination with two serotoninergic antidepressants.

## Case report

The patient was a 50-year-old male who presented with an episode of DTD lasting for several years. He was medically healthy and did not use any alcohol or recreational drugs. Since he had failed many trials of psychotropic medications (8 antidepressants, 3 atypical antipsychotics, 2 mood stabilizers and 1 psychostimulant), multiple years of psychotherapy, two courses of repetitive transcranial magnetic stimulation (30 treatments/course) and a course of intravenous ketamine (6 infusions), he was offered a trial of PAP through Health Canada’s Special Access Program. The 25 mg psilocybin was provided by Filament Health (Burnaby, Canada) and manufactured according to good manufacturing practice guidelines. The patient provided written consent for this case report publication.

**Figure 1:**
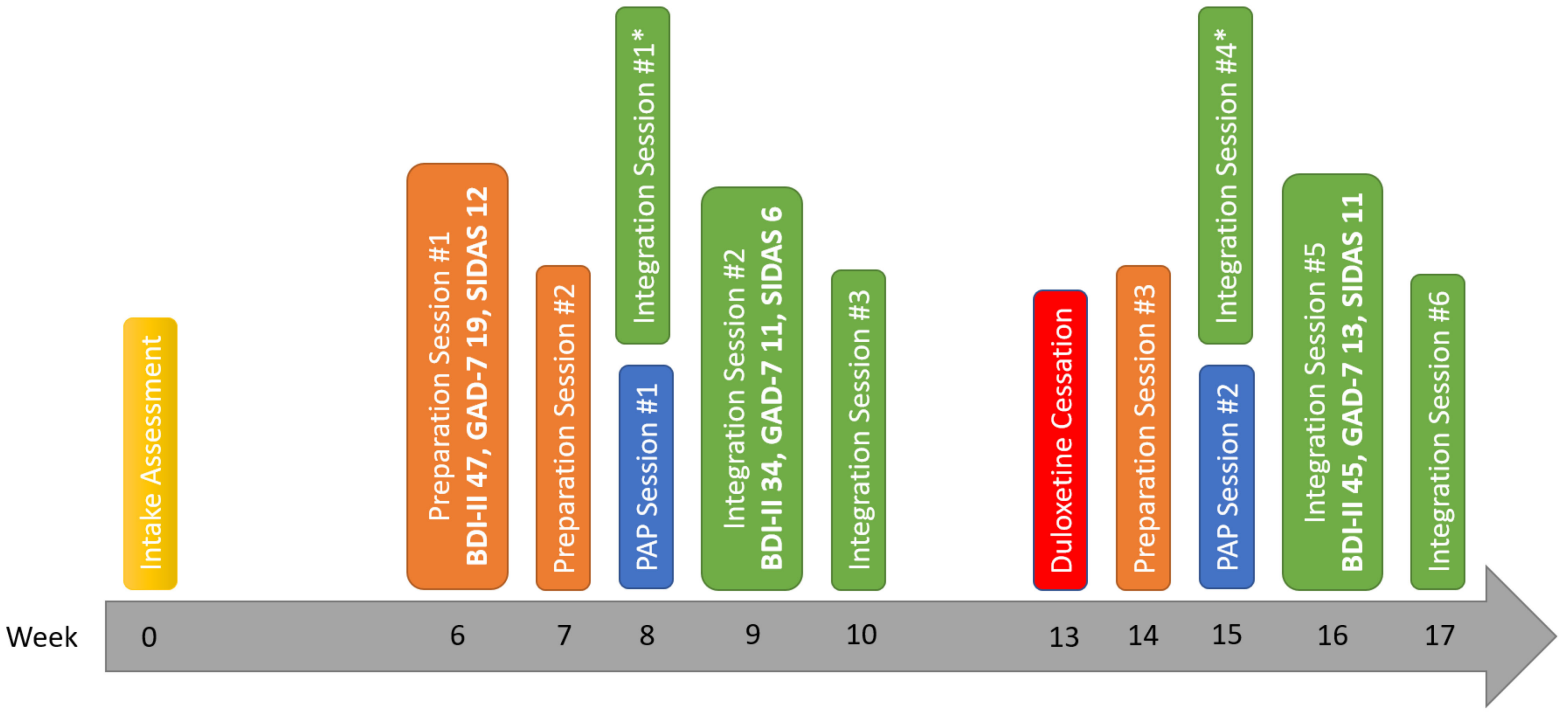
Timeline of significant events and rating scale scores. BDI-II, Beck Depression Inventory; GAD-7, Generalized Anxiety Disorder 7-Item; SIDAS, Suicidal Ideation Attributes Scale; PAP, Psilocybin-Assisted Psychotherapy. *Occurred the day after the PAP Session.

Before receiving PAP, the patient was taking duloxetine 60 mg DIE, vortioxetine 20 mg DIE, zolpidem 10-15 mg QHS PRN and quetiapine 25-50 mg DIE PRN. Given its potential to reduce psilocybin’s efficacy via 5-HT2A antagonism, he was told to hold quetiapine for one week prior to the PAP session. However, due to the highly refractory nature of his depression, the treating team decided collaboratively with the patient to maintain both antidepressants. During the PAP session, psilocybin was well-tolerated, with mild headaches being the only adverse effect reported by the patient. There was a mild increase in his heart rate (63 to 66) and blood pressure (119/77 to 141/93 mm Hg) at 90 minutes post-psilocybin ingestion. One week after the PAP session, his depression, anxiety and suicidality rating scale scores all improved from baseline; his Beck Depression Inventory (BDI-II) score decreased from 47 to 34, his Generalized Anxiety Disorder 7-Item (GAD-7) from 19 to 11 and his Suicidal Ideation Attributes Scale (SIDAS) from 12 to 6 (see **Figure 1**).

Given his partial response, the treating team offered the patient a second PAP session at the same dose, and discussed with him about discontinuing his antidepressants to see if it could lead to better treatment outcomes. The rationale for stopping his antidepressants was based on the hypothesis that they could attenuate the pharmacological effects of psilocybin via downregulation of the 5-HT2A receptors. He refused to discontinue vortioxetine, since he believed that it had been the most helpful medication for his depression so far, but agreed to stop duloxetine 2 weeks before the second PAP session. After stopping duloxetine, he experienced moderate discontinuation symptoms, including dizziness, muscle pain and fatigue.

The second PAP session occurred 7 weeks after the first one. Although he noted an improvement, he was still experiencing antidepressant discontinuation symptoms at the time of the second PAP session. Psilocybin was again well-tolerated, with no significant increase in his heart rate (59 to 61) and blood pressure (128/87 to 134/91 mm Hg). One week after the second PAP session, he experienced a worsening of his symptoms; his BDI-II score increased from 34 to 45, his GAD-7 from 11 to 13 and his SIDAS from 6 to 11.

## Discussion

We have presented the case of a patient with DTD, who initially received PAP in combination with two serotoninergic antidepressants (duloxetine and vortioxetine) and subsequently with one antidepressant (vortioxetine). Overall, psilocybin was well-tolerated; the patient only experienced mild headaches, and there were no cardiovascular safety concerns. Regarding effectiveness, psilocybin was originally associated with a reduction in depression, anxiety and suicidality, but his symptoms subsequently rebounded. We hypothesize that his clinical deterioration after the second PAP session may be explained by the persistence of his antidepressant discontinuation symptoms related to the abrupt cessation of duloxetine, as there were no other medication changes and no significant stressful life events between the two PAP sessions.

How antidepressant treatment impacts the therapeutic efficacy of psilocybin remains unclear. A recent online retrospective survey of individuals using psilocybin mushrooms found that concurrent selective serotonin reuptake inhibitors (SSRI)/serotonin and norepinephrine reuptake inhibitors (SNRI) use was associated with weaker acute psilocybin effects in approximately half of respondents (3). However, a small open-label study exploring the safety, tolerability and efficacy of a single 25 mg dose of psilocybin in participants taking a concomitant SSRI showed a generally favorable safety profile and antidepressant efficacy (4). Most treatment-emergent adverse effects were mild and transient, and the Montgomery-Åsberg Depression Rating Scale response rate was comparable to that of other recent, larger trials in which the participants were antidepressant-free. However, the study excluded participants taking multiple antidepressants. Although this case report suggests that 1) serotoninergic antidepressants combination with psilocybin may be safe and 2) antidepressant discontinuation prior to PAP may be unnecessary, a single case report should be interpreted with caution.

This case report raises several practical points. Despite the theoretical risk of serotonin syndrome, the combination of serotoninergic antidepressants and psilocybin appears to be safe. In addition, real-world patients with DTD are likely to be on a combination of psychotropic medications and may be reluctant to stop them, especially if they perceive a benefit from their regimen. Finally, SSRI/SNRI can be associated with antidepressant discontinuation symptoms. Although they are generally mild and short-lived, there is evidence suggesting that antidepressant discontinuation symptoms can be more severe and persist longer over time. The continuation of antidepressants during PAP has the potential to improve treatment acceptability and accessibility for patients who want to stay on their current medication regimen, those at risk for antidepressant discontinuation symptoms and those at high risk of relapse. Therefore, whether psilocybin can be administered concurrently with antidepressants is an important research question, and larger, randomized-controlled trials are needed.

## Data availability statement

The original contributions presented in the study are included in the article/supplementary material. Further inquiries can be directed to the corresponding author.

## Ethics statement

Written informed consent was obtained from the individual(s) for the publication of any potentially identifiable images or data included in this article.

## Author contributions

AD: Writing – original draft, Writing – review & editing. VM: Writing – review & editing. J-FS: Writing – review & editing. MM: Writing – review & editing. ÉB: Writing – review & editing. F-AB: Writing – review & editing. AB-DS: Writing – review & editing. AT: Writing – review & editing. PV: Writing – review & editing.

## References

[1] Goodwin GM Aaronson ST Alvarez O Arden PC Baker A Bennett JC. Single-dose psilocybin for a treatment-resistant episode of major depression. New Engl J Med. (2022) 387:1637–48. doi: 10.1056/NEJMoa2206443 36322843

[2] Sarparast A Thomas K Malcolm B Stauffer CS. Drug-drug interactions between psychiatric medications and MDMA or psilocybin: a systematic review. Psychopharmacology. (2022) 239:1945–76. doi: 10.1007/s00213-022-06083-y PMC9177763 35253070

[3] Gukasyan N Griffiths RR Yaden DB Antoine DG 2nd Nayak SM. Attenuation of psilocybin mushroom effects during and after SSRI/SNRI antidepressant use. J Psychopharmacol (Oxford England). (2023) 37:707–16. doi: 10.1177/02698811231179910 37291890

[4] Goodwin GM Croal M Feifel D Kelly JR Marwood L Mistry S. Psilocybin for treatment resistant depression in patients taking a concomitant SSRI medication. Neuropsychopharmacology: Off Publ Am Coll Neuropsychopharmacol. (2023) 48:1492–9. doi: 10.1038/s41386-023-01648-7 PMC10425429 37443386

